# Transnasal, Transethmoidal Endoscopic Removal of a Foreign Body in the Medial Extraconal Orbital Space

**DOI:** 10.1155/2016/1981456

**Published:** 2016-11-13

**Authors:** Diego Escobar Montatixe, José Miguel Villacampa Aubá, Álvaro Sánchez Barrueco, Beatriz Sobrino Guijarro, Carlos Cenjor Español

**Affiliations:** ^1^Department of Otolaryngology, Head and Neck Surgery, Hospital Universitario Fundación Jiménez Díaz, Madrid, Spain; ^2^Department of Neuroradiology, Hospital Universitario Fundación Jiménez Díaz, Madrid, Spain

## Abstract

Intraorbital foreign bodies are located within the orbit but outside the ocular globe. Though not uncommon, removal of these objects poses a challenge for surgeons. External approaches have been the most frequently used but are associated with increased complications and morbidity. An endoscopic endonasal approach can be an appropriate and less complicated technique in these cases. We report a case of a chronic intraorbital foreign body located within the medial extraconal space lateral to the lamina papyracea and behind the lacrimonasal duct, which was successfully removed using a transnasal, transethmoidal endoscopic technique. Neither postoperative complications nor ocular impairment was reported. The patient improved and remains asymptomatic. The transnasal transethmoidal endoscopic approach can be used as a safer and less invasive alternative when removing foreign bodies from the medial orbital compartment.

## 1. Introduction

Intraorbital foreign bodies are rare and pose a challenge for surgeons. The term “intraorbital” refers to those foreign bodies located within the orbit but outside the ocular globe [1]. Classically, external approaches have been the most widely used; however, these are invasive and associated with several major disadvantages such as postsurgical scarring and considerable morbidity. As technology and the understanding of the anatomy gradually progress, surgeons are using endoscopic techniques for diseases located outside the nasal sinuses.

The medial portion of the orbit shares a boundary with the ethmoid and sphenoid sinuses, and surgeons have exploited this proximity to access the orbit [2]. Foreign bodies located close to the medial wall of the orbit can be safely removed using an endoscopic transnasal approach [3]. This technique is less invasive and, when performed by an experienced surgeon, is associated with fewer complications. We present the case of a female patient with a chronic intraorbital foreign body (a shard of glass) located within the medial extraconal space and successful removal of the body using a transnasal, transethmoidal endoscopic approach.

## 2. Case Report

**Figure 1:**
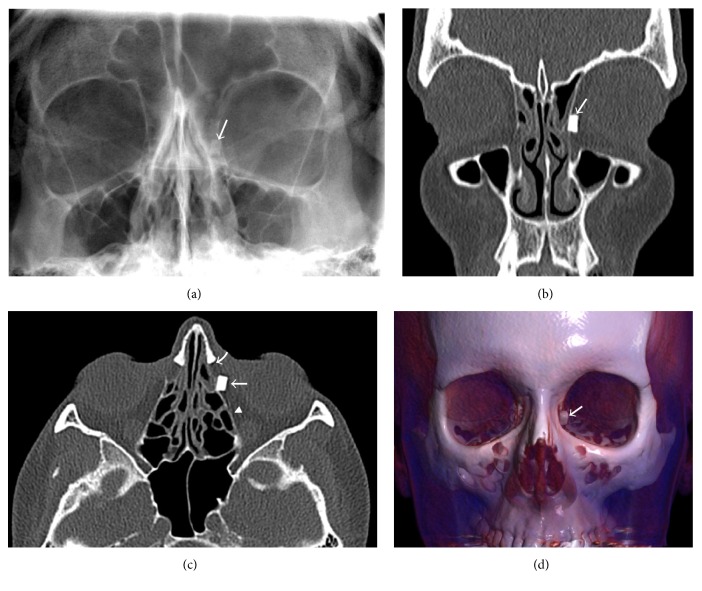
(a–d) Frontal plain radiograph revealing (a) a rectangular high-density body (arrow) overlying the left ethmoid cells and in contact with the lamina papyracea. Contrast unenhanced axial (b) and coronal (c) CT scans show a well-delineated hyperdense body (arrow) within the left orbit, adjacent to the lamina papyracea (arrowhead in (c)) and immediately posterior to the lacrimonasal duct (curved arrow in (c)). (d) 3D-CT volume-rendered reconstructed image shows the intraorbital metallic foreign body (arrow).

A 41-year-old woman presented to our department with a 2-year history of recurrent left orbital edema and erythema which had partially improved with oral antibiotics. The patient also reported mucopurulent rhinorrhea and intermittent bilateral nasal stuffiness not related to the orbital symptomatology. Relevant past history was limited to a road traffic accident approximately 5 years previously; following this accident, a residual foreign body (glass) located medially to the left eye globe was detected on a plain radiograph performed at the time (Figure 1(a)). The patient initially declined to undergo a foreign body removal procedure, although persistence of chronic symptomatology led her to consult with our department on available surgical options.

Nasal endoscopy detected hypertrophy of the left middle turbinate and an accompanying mucopurulent discharge from the middle meatus, without other relevant findings.

A computerized tomography (CT) scan of the facial and paranasal sinuses showed slight middle meatal stenosis due to a bullous left middle turbinate with normal, air-filled sinus cavities and the presence of a hyperdense, well-defined foreign body (a shard of glass) located within the eye orbit in the medial extraconal space, laterally to the lamina papyracea and posteriorly to the nasolacrimal duct (Figures 1(b), 1(c), and 1(d)).

**Figure 2:**
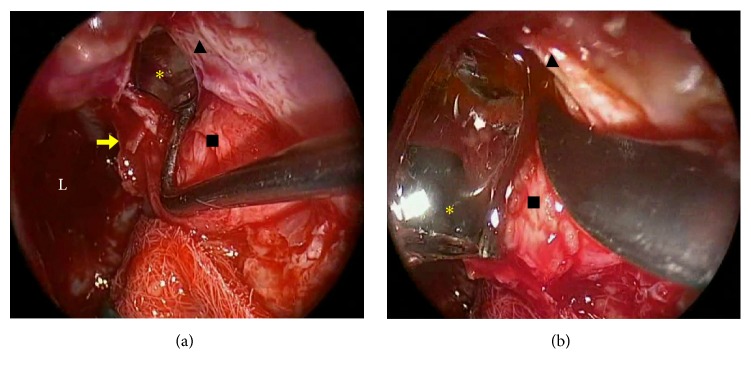
(a-b) Left transnasal endoscopic view of a shard of glass (yellow asterisk) following the aperture of the lamina papyracea (yellow arrow) and left periorbital fat (black square) immediately behind the nasolacrimal duct (black arrowhead).

At the request of the patient, we began planning to remove the foreign body endoscopically using a transnasal transethmoidal approach accompanied by a widening of the osteomeatal complex as treatment of her middle meatal stenosis. Partial resections of the bullous middle turbinate, maxillary antrostomy, and anterior ethmoidectomy with aperture of the left lamina papyracea were performed; the foreign body was located within the periorbital fat and was successfully removed without evidence of lesions to the extraocular musculature or the nasolacrimal duct (Figure 2). No ocular movement anomalies or other immediate postsurgical complications were detected. On follow-up, the patient remains asymptomatic, presenting no additional episodes of left orbital cellulitis to date.

## 3. Discussion

From an anatomic point of view, the orbit is a highly complex area where critical structures occupy a small space [4]. The cone formed by the extraocular muscles divides the orbit into two compartments: intraconal and extraconal [5]. Intraorbital foreign bodies are located in the orbit and the vast majority are secondary to facial trauma involving penetration of the orbit. The surgical approach employed for extraction depends on the nature of the body, its location (anterior or posterior orbit), and associated complications (infections, optic nerve lesions or compression, and lesions to the extraocular nerve or intraorbital blood vessels) [5].

Complex craniomaxillofacial, transethmoidal, or transcranial approaches have traditionally been used to reach the invading material, especially when foreign bodies are located in the medial aspect of the orbit [6, 7]. However, external approaches require skin incisions, osteotomies, and significant displacement of orbital structures, including the globe. The endoscopic endonasal technique can be considered a surgical option to manage the optic nerve and orbital compartments (medial side) for various posttraumatic, inflammatory, infectious, or tumoral diseases; moreover, it minimizes external scarring and preserves cosmesis [8]. From an anatomic viewpoint, this procedure appears to be an excellent surgical approach to access the medial compartment of the orbit and the orbital apex [9]. This alternative should be taken into consideration during surgical planning, especially when dealing with medial extra- and intraconal orbital lesions, including foreign bodies located close to the medial wall of the orbit [3, 8].

Neuronavigation has been shown to be an essential element in endoscopic intervention within the orbit, as it allows for precise location of the target, thereby enabling surgeons to make the smallest possible opening in the bone and periorbita [10–12]; in our patient, however, as the intraorbital foreign body was located in such a concrete and accessible location (beside the lamina papyracea and behind the nasolacrimal duct), we opted not to use this implement. Therefore, we performed a transnasal transethmoidal endoscopic approach to access the lamina papyracea, which preserved our anatomic references (the nasolacrimal duct in this case), thus making this a less invasive approach and a feasible alternative.

## 4. Conclusion

The transnasal transethmoidal endoscopic approach can be employed as an alternative when removing intraorbital foreign bodies located in the medial extraconal compartment. This is a safe and less invasive approach in comparison with classic surgical techniques.

## References

[1] Fulcher T. P., McNab A. A., Sullivan T. J. (2002). Clinical features and management of intraorbital foreign bodies. *Ophthalmology*.

[2] Ramakrishnan V. R., Suh J. D., Chiu A. G., Palmer J. N. (2011). Addition of a minimally invasive medial orbital approach in the endoscopic management of advanced sino-orbital disease: cadaver study with clinical correlations. *Laryngoscope*.

[3] Łysoń T., Sieskiewicz A., Rogowski M., Mariak Z. (2013). Transnasal endoscopic removal of intraorbital wooden foreign body. *Journal of Neurological Surgery, Part A: Central European Neurosurgery*.

[4] Dallan I., Seccia V., Lenzi R. (2010). Transnasal approach to the medial intraconal space: anatomic study and clinical considerations. *Minimally Invasive Neurosurgery*.

[5] Turliuc D. M. I., Costan V. V., Cucu A. I., Costea C. F. L. (2015). Intraorbital foreign body. *Revista medico-chirurgicala a Societatii de Medici si Naturalisti din Iasi*.

[6] Edgington B. D., Geist C. E., Kuo J. (2008). Intraorbital organic foreign body in a tree surgeon. *Ophthalmic Plastic and Reconstructive Surgery*.

[7] Feichtinger M., Zemann W., Kärcher H. (2007). Removal of a pellet from the left orbital cavity by image-guided endoscopic navigation. *International Journal of Oral and Maxillofacial Surgery*.

[8] Castelnuovo P., Turri-Zanoni M., Battaglia P., Locatelli D., Dallan I. (2015). Endoscopic endonasal management of orbital pathologies. *Neurosurgery Clinics of North America*.

[9] Brown S. M., Schwartz T. H., Anand V. K. (2007). The transethmoidal, transorbital approach to the orbital apex. *Practical Endoscopic Skull Base Surgery*.

[10] Kent J. S., Allen L. H., Rotenberg B. W. (2010). Image-guided transnasal endoscopic techniques in the management of orbital disease. *Orbit*.

[11] Schramm A., Suarez-Cunqueiro M. M., Rücker M. (2009). Computer-assisted therapy in orbital and mid-facial reconstructions. *The International Journal of Medical Robotics and Computer Assisted Surgery*.

[12] Sieskiewicz A., Lyson T., Mariak Z., Rogowski M. (2008). Endoscopic trans-nasal approach for biopsy of orbital tumours using image-guided neuro-navigation system. *Acta Neurochirurgica*.

